# Supplementary material for: Mycetoma: A critical appraisal of educational content on YouTube
Source: PLoS Negl Trop Dis. 2024 Nov 11;18(11):e0012660. doi: 10.1371/journal.pntd.0012660 (PMC11623468; doi:10.1371/journal.pntd.0012660)
Supplement: Attachment — Submitted filename: PNTD-D-24-01274- Pitua_R2R[1].docx [file pntd.0012660.s002.docx]

Ahmed Hassan Fahal, FRCS, FRCSI, FRCSG, MS, MD, FRCP(London)
Academic Editor
PLOS Neglected Tropical Diseases

Dear Prof Fahal,

Many thanks for editing this work.

We have provided point by point responses to the reviewers’ comments and hope this work is now acceptable.

Kind regards,

**Dr Felix Bongomin**

**PNTD-D-24-01274: Mycetoma: Critical Appraisal of Educational Content on YouTube**

Reviewer #1: 3.	Methods: The 3 assessors – are they experienced in the clinical or laboratory aspects of mycetoma? Are they doctors? All presentations need to address particular audiences – so what audience are they?

Authors’ response: We appreciate the reviewer’s query. All three are medical doctors with significant experience in clinical research and the laboratory diagnosis of fungal diseases, including mycetoma. The 3 assessors have closely worked with Dr. Felix Bongomin in his mycetoma studies in Northern Uganda and have gone a series of rigorous trainings in that aspect. Healthcare workers as well as the general public are being addressed.

4.	Needs more clarification of DISCERN tool and Global Quality Score – what are the metrics for these results? What is being measured?
Authors’ response: Thank you. We have now added more details in the methods section to clarify the DISCERN tool and the Global Quality Score (GQS).
Reviewer #2: - It might be helpful to add why videos with less than 100 views were excluded? Is this standard based on past research in video evaluation?

Authors’ response: Yes, this was based on previously described methodology – now added in the methods section.
- Consider including how researchers distinguished between consumer and professional groups who uploaded the videos
Authors’ response: Thank you for these. These have been clarified and additional supplementatry material adde.
Reviewer #3: The methodology is well laid out.
Authors’ response: Thank you.

Reviewer #1: 5.	Results: is there any data or correlation between the source country and quality? The WHO Mycetoma Collaborating centre is unique. Could these videos be regarded as the ‘highest quality benchmark’ against which to judge others?
Authors’ response: Thank you for the insightful suggestion. While we did not specifically analyze the source country in our current study due to limitation of the R code, we acknowledge that the WHO Mycetoma Collaborating Centre’s videos could serve as a benchmark for quality but it is important to note we cannot categorically state that all videos from MRCs scored “Highly” when subjected to the tools as some videos still lacked the precision explored by the scoring tools.
6.	Presumably with a median duration of 8 minutes, it was impossible for the shorter videos to address clinical presentation, diagnosis and therapy. So how did the these categories of video perform on the quality scores?
Authors’ response: We agree with the reviewer that shorter videos may not comprehensively cover all aspects of clinical presentation, diagnosis, and therapy. Our analysis indicated that shorter videos generally received lower Global Quality Scores and DISCERN scores, reflecting their limited scope. This is discussed further in the manuscript under the results as well as the discussion section.

7.	Can the authors make any general comments about what makes a good video for mycetoma? Clinical photographs? Easily readable slides? Demonstration of diagnostic procedure(s)? Pace of presentation? Depth of data? The reader has no real sense of what the scores are really measuring?
Authors’ response: We focused video quality/reliability based on the components of the mDISCERN and GQS tools. It covers all aspects, this has been detailed in the new Table 1.
8.	The paper should have the listing of all videos scored as a supplementary excel file. Others in the future may wish to address this.
Authors’ response: Thank you for the suggestion. We have now provided the full list of videos evaluated, as a supplementary Excel file.

Reviewer #2: Consider putting the actual range of the IQR which may be easier to comprehend quickly for the reader. Right now, it seems like only the upper range is included.
Authors’ response: We have now included both the lower and upper ranges of the interquartile ranges (IQR) in the results section to enhance clarity.
Reviewer #3: The results are clear.
Although the results are interesting, more information on the characteristics of the videos is needed. I suggest  include, years, country of publication, type of source (media, user-generated content, health professionals and others) and publication of the videos (news, material created by the user, interviews, advertisements and documentaries).
Authors’ response: The R code is unable to discern all these, it can only extract YouTube matrices, and also our criteria excluded adverts/commercials. We found it more inclusive to have a binary grouping (i.e. Professional Vs Consumer group) for a less burdensome analysis based on reports from previous literature. We opt to indicate this in limitations.
It is also important to consider information related to the mycetoma discussed in the videos. For example, recommendation for healthcare workers, recommendation for all persons over 18 years of age 15, recommendation for persons 60 years of age or older, diagnosis, treatment, etc.
Authors’ response: As above, our study was limited to assessment of reliability and quality and so the in depth data on all these aspects were not extracted as per the scope of the review. This has been hinted in the limitations.
Reviewer #1: Lack of clarity about defines quality
Authors’ response: This has been detailed in Table 1 as aspects of the GQS tool.
Reviewer #2: - As in introduction, would consider putting some caveats in about actual rise in prevalence of mycetoma rather than just increased reporting due to awareness and diagnostic access.

Authors’ response: This has been covered.
- Would move the reasoning for excluding videos based on length into methodology section.

Authors’ response: This has been covered.
- While not systemically evaluated, it could be interesting to touch upon the scientific content that was included (diagnosing, symptoms, etc) and the gaps the authors found in terms of actual content.

Authors’ response: Noted and hinted in discussion.
- Might be interesting to include some discussion about digital misinformation and the impact on populations trust in medical and public health institutions and how this could impact mycetoma with false information on youtube.
Authors’ response: Noted and hinted in discussion.
Reviewer #3: Discussion. Include a paragraph with the implications of your findings for public health.

Done as required.
Reviewer #1: Comments
1.	Ref 1 is not a good general reference. There are other reviews, including Ref 3.

Authors’ response: We have revised this to feature a WHO source and additional epidemiological references.
2.	Ref 3 and 4 about the epidemiology and ‘Mycetoma belt ignore the most recent paper of global distribution: <https://doi.org/10.1371/journal.pntd.0008397>
Authors’ response: We have added the above most recent paper by Emery and Denning as the source, content is essentially the same.
Reviewer #2: N/A

Reviewer #3: None

Reviewer #1: This is an interesting topic that has not previously seen much research. YouTube as an educational tool in this field has not been analysed, so the study is novel. The study is well written and results are easy to understand.
Authors’ response: Thank you so much.

Reviewer #2: Thank you so much for the opportunity to review this article examining the quality of mycetoma content on Youtube. The authors take a unique approach to evaluating public health and clinical content on mycetoma, and this article could be really interesting in the context of medical and public health disinformation. 
Authors’ response: Thank you so much for your Kind words.
A few considerations for the authors are below: 
- Line 51: It is difficult to state the the prevalence of mycetoma is increasing rather than just being reported more often due to raised awareness and access to diagnostics. With very few countries having surveillance systems, consider modifying this language to reflect that cases are being reported more often.
Authors’ response: We agree. We have revised this in part of the introduction and part of discussion.

Reviewer #3: In this study aimed at exploring these aspects of YouTube videos on mycetoma. Findings show mycetoma-related videos on YouTube are generally of high quality, with moderate reliability. 
Authors’ response: Exactly !.
Although the study design and results are well described. Information for prevention, diagnosis, management etc. is needed. These recommendations are important for public health and tropical diseases. 
Authors’ response: Noted and hinted in introduction and discussion.
Comments: 
Although the results are interesting, more information on the characteristics of the videos is needed. I suggest  include, years, country of publication, type of source (media, user-generated content, health professionals and others) and publication of the videos (news, material created by the user, interviews, advertisements and documentaries).
Authors’ response: The R code is unable to discern all these, it can only extract YouTube matrices, and also our criteria excluded adverts/commercials. We found it more inclusive to have a binary grouping (i.e. Professional Vs Consumer group) for a less burdensome analysis based on reports from previous literature. We opt to indicate this in limitations.
It is also important to consider information related to the mycetoma discussed in the videos. For example, recommendation for healthcare workers, recommendation for all persons over 18 years of age 15, recommendation for persons 60 years of age or older, diagnosis, treatment, etc

Authors’ response: As above, our study was limited to assessment of reliability and quality and so the in depth data on all these aspects were not extracted as per the scope of the review. This has been hinted in the limitations.
